# Two New Triterpenoids from the Roots of *Codonopsis pilosula*

**DOI:** 10.3390/molecules23020383

**Published:** 2018-02-11

**Authors:** Tao Zheng, Li-Zhi Cheng, Yong-Ming Yan, Bao-Hua Liu, Fu-Ying Qin, Fu-Rong Xu, Yong-Xian Cheng

**Affiliations:** 1 College of Pharmacy, Yunnan University of Traditional Chinese Medicine, Kunming 650504, China; zhengtao@cuhk.edu.hk (T.Z.); qinfuying@mail.kib.ac.cn (F.-Y.Q.); 2 State Key Laboratory of Phytochemistry and Plant Resources in West China, Kunming Institute of Botany, Chinese Academy of Sciences, Kunming 650201, China; 3 Guangdong Key Laboratory for Genome Stability & Disease Prevention, School of Pharmaceutical Sciences, Shenzhen University Health Sciences Center, Shenzhen 518060, China; 13424039397@163.com (L.-Z.C.); yanym@szu.edu.cn (Y.-M.Y.); ppliew@szu.edu.cn (B.-H.L.); 4 College of Pharmacy, Henan University of Chinese Medicine, Zhengzhou 450008, China

**Keywords:** *Codonopsis pilosula*, pseudolarolides, triterpenoids, SIRT1

## Abstract

Pseudolarolides U and V, two new triterpenoids, and four biogenetically related compounds, pseudolarolides E, F, K, and P were isolated from the roots of *Codonopsis pilosula* (Campanulaceae). Their structures were determined by spectroscopic data. The regulation of Sirtuin 1 (SIRT1) activity by all the isolated compounds was evaluated.

## 1. Introduction

**Figure 1:**
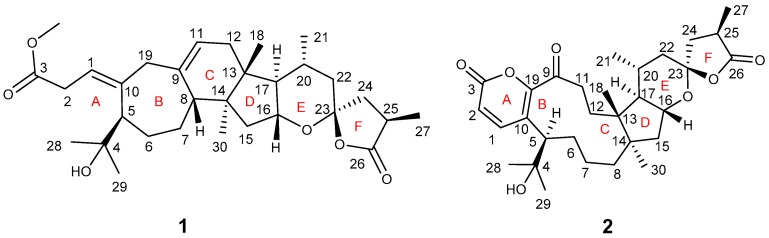
The structures of compound **1** and compound **2** from *Codonopsis pilosula.*

The root of *Codonopsis pilosula*, known as “Dangshen” in Chinese, has been used in traditional Chinese medicine to treat “qi” deficiency, improve appetite, and strengthen the immune system [1,2]. Previous phytochemical research on this species revealed that *C. pilosula* contains polyacetylenes, phenylpropanoids, alkaloids, triterpenoids, and polysaccharides [3,4,5,6], which possess multiple biological activities, such as immunity regulation, learning and memory improvement, and nitric oxide inhibition [7,8]. We investigated the roots of *C. pilosula* and compared the chemical profile of the species produced in Yunnan and Shanxi provinces. During our efforts, six triterpenoidal lactones including two new compounds (**1** and **2**) (Figure 1), and four known triterpenoids, pseudolarolides E (**3**), F (**4**), K (**5**), and P (**6**), [9,10,11], were isolated. Considering that the roots of *C. pilosula* have, as mentioned above, tonic effects, and that Sirtuin 1 (SIRT1) is associated with many diseases also related to ageing, the biological evaluation of all the isolated compounds with respect to SIRT1 was conducted [12,13]. In this contribution, we describe the isolation, structural characterization, and biological evaluation of the mentioned compounds.

## 2. Results and Discussion

### 2.1. Elucidation of the Compounds

The EtOH extract of *C. pilosula* roots was suspended in water and subsequently partitioned with petroleum ether and EtOAc. A combination of column chromatorgaphy on the EtOAc extract afforded compounds **1**–**6**.

**Figure 2:**
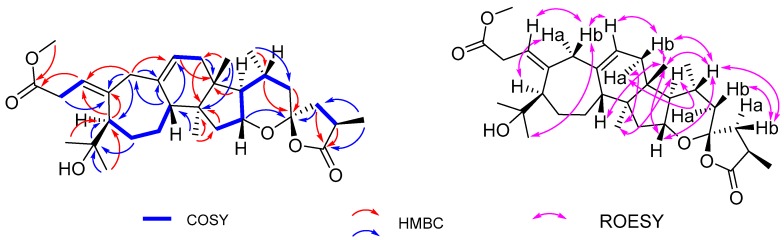
Key COSY, HMBC, and ROESY correlations for compound **1.**

Compound **1**, obtained as a yellow gum, had the molecular formula C_31_H_46_O_6_ (9 degrees of unsaturation), based on the analysis of its HRESIMS, ^13^C-NMR, and DEPT spectra. The ^1^H-NMR spectrum of **1** (Table 1) indicated the presence of seven methyl groups (*δ*_H_ 3.61 (3H, s, OCH_3_), 1.22 (3H, d, *J* = 7.3 Hz, H_3_-27), 1.18 (3H, s, H_3_-29), 1.12 (3H, s, H_3_-28), 0.88 (3H, d, *J* = 6.2 Hz, H_3_-21), 0.83 (3H, s, H_3_-30), and 0.70 (3H, s, H_3_-18)) and two olefinic protons (*δ*_H_ 5.63 (1H, t, *J* = 7.7 Hz, H-1), 5.19 (1H, t, *J* = 5.3 Hz, H-11)). The ^13^C-NMR and DEPT spectra showed that this substance contained 31 carbons including 7 methyl, 8 methylene, 8 methine (two sp^2^, six sp^3^), and 8 quaternary carbons (4 sp^2^ including 2 carboxyls, 4 sp^3^). These data suggested that compound **1** might be a triterpenoid. A detailed analysis of the ^1^H- and ^13^C-NMR data (Table 1) found that **1** is similar to pseudolarolide O [10]. The difference is that a lactone ring formed by C_3_–O–C_4_ in pseudolarolide O is broken in **1**. This was supported by the characteristic chemical shift of C-4 (*δ*_C_ 81.6 for pseudolarolide O and *δ*_C_ 73.2 in **1**). The HMBC correlation of OMe/C-3(*δ*_C_ 173.0) confirmed the location of the methoxy group (Figure 2). The relative configuration of **1** was assigned by ROESY data. The ROESY spectrum showed correlations of Ha-19/H-5, Hb-19/H-29, H-11, H-11/Hb-12, Ha-12/H-18, H-20, H-18/H-8, H-20, H-16, H-16/H-20, which led to the assignment of the relative configurations of rings B–E. There is one chiral carbon in ring F whose relative configuration is rather difficult to assign. Because it was not possible to get a crystal for X-ray diffraction analysis, the absolute configuration of C-25 was tentatively postulated to be the same as that of pseudolarolide O from a biogenetic point of view. Taken together, the structure of **1** was elucidated and named pseudolarolide U.

Compound **2** was obtained as a white solid. Its molecular formula, C_30_H_42_O_7_, was determined by means of HRESIMS, ^13^C-NMR, and DEPT spectra as having 10 degrees of unsaturation. The ^1^H-NMR spectroscopic data of **2** (Table 1) revealed the presence of six single methyl (*δ*_H_ 1.01 (d, *J* = 6.5 Hz, H_3_-21), 1.23 (d, *J* = 7.2 Hz, H_3_-27), 0.78 (s, H_3_-18), 1.05 (s, H_3_-28), 1.29 (s, H_3_-29), 1.31 (s, H_3_-30)) and two olefinic protons (*δ*_H_ 6.24 (1H, d, *J* = 9.7 Hz, H-2), 7.41 (1H, d, *J* = 9.7 Hz, H-1)). The ^13^C-NMR and DEPT spectra (Table 1) showed 30 carbons ascribed to 6 methyl, 8 methylene, 7 methine (2 sp^2^ and 5 sp^3^), and 9 quaternary carbons (1 ketone, 2 olefinic including 1 oxygenated, 2 carbonyls and 4 aliphatic). These data are similar to those of pseudolarolide P [10], suggesting that the two compounds are analogues. 

**Figure 3:**
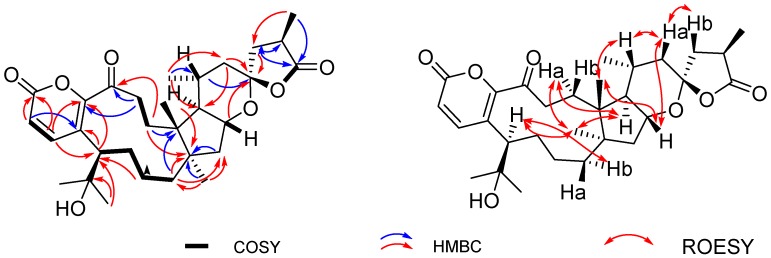
Key COSY, HMBC, and ROESY correlations for compound **2**.

One difference between **2** and pseudolarolide P is that the *Δ*^5(6)^ double bond in pseudolarolide P is absent in **2**, as supported by the ^1^H-^1^H COSY correlations of H-5/H-6/H-7/H-8 (Figure 3). In addition, ring C in pseudolarolide P disappeared revealing, instead, the presence of a ketone (C-9), which could be evidenced by the HMBC correlations of H-11, H-12/C-9 (*δ*_C_ 201.2). Finally, it was found that the chemical shift of C-4 (*δ*_C_ 79.0) in pseudolarolide P is upshifted in **2** (*δ*_C_ 73.2 for C-4), indicating the cleavage of C(3)-O-C(4) in pseudolarolide P and the presence of a free OH group at C-4 in **2**. The chemical shift for C-19 (*δ*_C_ 159.9), the HMBC correlations of H-5, H-11/C-19, and the degree of unsaturation for **2** indicated, as the only possibility, the formation of C(3)-O-C(19). In this way, the planar structure of **2** was deduced as shown. As for the relative configuration for **2**, it might be the same as that of pseudolarolide P. The ROESY correlations of H-30/H-5, H-17, H-16/H-18, Ha-22, H-20/Ha-22, H-18 indicated that these protons are adjacent to each other. In the same manner as for **1**, the relative configuration of ring F was presumed to be the same as that of the other reported pseudolarolides, from a biogenetic point of view [9,10,14,15,16,17,18]. As a result, the structure of **2** was established and named pseudolarolide V.

Four known compounds were identified as pseudolarolide K (**3**) [11], pseudolarolide P (**4**) [10], pseudolarolide E (**5**) [9], and pseudolarolide F (**6**) [9] by comparison of their spectroscopic data with literature data.

### 2.2. Biological Evaluation

SIRT1 is a nicotinamideadenosine dinucleotide (NAD)-dependent deacetylase regulating a variety of cellular functions, including cellular stress responses and energy metabolism [19,20]. Using a specific assay, all the isolates were tested for their regulatory activity of SIRT1, with nicotinamide as a positive control. Unfortunately, all the compounds were not active at a concentration of 200 μM (data not shown). 

## 3. Experimental Section

### 3.1. General Procedures

The optical rotation was recorded by a Bellingham+ Stanley ADP 440+ digital polarimeter (Bellingham & Stanley, Kent, UK). UV spectra were collected on a Shimadzu UV-2401PC spectrometer (Shimadzu Corporation, Tokyo, Japan). NMR spectra were measured via a Bruker AV-400 MHzor, a Bruker Avance III 600 MHz spectrometer (Bruker, Karlsruhe, Germany), TMS was used as an internal standard. ESIMS and HRESIMS were obtained on an API QSTAR Pulsar 1 spectrometer (Applied Biosystems, MDS Sciex, Framingham, MA, USA). RP-18 (40−60 μm; Daiso Co., Tokyo, Japan), silica gel (200–300 mesh; Qingdao Marine Chemical Inc., Qingdao, China), MCI gel CHP 20P (75−150 μm, Mitsubishi Chemical Industries, Tokyo, Japan), and Sephadex LH-20 (Amersham Pharmacia, Uppsala, Sweden) were used for column chromatography. Semipreparative HPLC was carried out using a LC-3000 liquid chromatograph equipped with an Agilent Zorbax SB-C18 column (250 mm × 9.4 mm, i.d., 5 μm) (Agilent Technologies, Santa Clara, CA, USA).

### 3.2. Plant Material

The dried roots of *C. pilosula* were supplied by the planting base of Shanxi Zhendong Pharmaceutical Company, in December 2015. A voucher specimen (CHYX-0598) was deposited at the State Key Laboratory of Phytochemistry and Plant Resource in West China, Kunming Institute of Botany, Chinese Academy of Science, Kunming, China.

### 3.3. Extraction and Isolation

The roots of *C. pilosula* (16.0 kg) were powdered and extracted under reflux with 80% EtOH (3 × 40 L × 1 h) to give a crude extract, which was suspended in water, followed by successive partition with petroleum ether and EtOAc to afford an EtOAc soluble extract (220 g). This extract was divided into nine parts (Frs. A–I) by a MCI gel CHP 20P column eluted with aqueous MeOH (5–100%), of which Fr. H (8.0 g) was subjected to column chromatography on silica gel eluted with gradient CHCl_3_/MeOH (14:1, 10:1, 6:1, 3:1, 0:1) to give five fractions (Frs. H.1−H.5). Among these, Fr. H.3 (2.5 g) was purified by Sephadex LH-20 (MeOH), followed by semipreparative HPLC (CH_3_CN/H_2_O, 75%) to yield compounds **3** (t_R_ = 15.0 min, 5.5 mg) and **6** (t_R_ = 18.2 min, 6.2 mg,). Fr. I (20.0 g) was subjected to column chromatography on silica gel eluted with a gradient of petroleum ether/acetone (14:1, 10:1, 6:1, 3:1, 0:1) to give 10 fractions (Frs. I.1−I.10), of which Fr. I.8 (5.5 g) was gel-filtrated over Sephadex-LH20 (MeOH) to produce three fractions (Frs. I.8.1−I.8.3). Fr. I.8.1 (205 mg) was purified by semipreparative HPLC (CH_3_CN/H_2_O, 75%) to yield compound **1** (t_R_ = 15.1 min, 1.8 mg). Fr. I.10 (1.5 g) was further separated by Sephadex LH-20 (MeOH) to yield two fractions (Frs. I.10.1 and I.10.2). Fr. I.10.2 (120 mg) was submitted to repeated semipreparative HPLC (CH_3_CN/H_2_O, 78%) to get compound **5** (t_R_ = 17.8 min, 2.4 mg). Fr. I.10.1 (150 mg) was subjected to column chromatography on silica gel eluted with CHCl_3_/MeOH (15:1) to yield compounds **2** (1.5 mg) and **4** (5.8 mg)

### 3.4. Compound Characterization Data

Pseudolarolide U (**1**): Yellowish gum; $\alpha_{D}^{20.7}$ + 2.4 (*c* 0.09, MeOH); UV (MeOH) *λ*_max_ (log*ε*): 203 (3.84) nm; ^1^H- and ^13^C-NMR data, see Table 1; ESIMS *m*/*z* 537 [M + Na]^+^; HRESIMS *m*/*z* 537.3183 [M + Na]^+^ (calcd. for C_3__1_H_4__6_NaO_6_, 537.3187). 

Pseudolarolide V (**2**)*:* Yellowish gum; $\alpha_{D}^{21.3}$ + 2.6 (*c* 0.15, MeOH); UV (MeOH) *λ*_max_ (log*ε*): 303 (3.76), 265 (3.80), 202 (3.74) nm; ^1^H- and ^13^C-NMR data, see Table 1; ESIMS *m*/*z* 537 [M + Na]^+^; HRESIMS *m*/*z* 537.2821 [M + Na]^+^ (calcd. for C_30_H_42_NaO_7_, 537.2823). 

### 3.5. SIRT1 Inhibition

The SIRT1 inhibitory activity of the compounds was screened using a fluorescence-based deacetylase assay [21]. In detail, each well consisted of 0.5 U (1 U = 1 pmol/min at 37 °C) of SIRT1 enzyme, 1000 μM of NAD^+^ (Enzo Life Sciences, Farmingdale, NY, USA), 100 μM of SIRT1 peptide substrate (Enzo Life Sciences), and SIRT1 assay buffer (50 mM Tris-HCl, pH 8.0, 137 mM NaCl, 2.7 mM KCl, 1 mM MgCl**_2_**, 1 mg/mL BSA) along with the test compound at indicated concentration. The plate was incubated at 37 °C for 30 min, and the reaction was stopped using Fluor de Lys developer II solution (Enzo Life Sciences) containing 2 mM nicotinamide. The plate was further incubated at 37 °C for additional 30 min, and the samples were read by a fluorimeter with an excitation wavelength of 360 nm and an emission wavelength of 460 nm. 

## 4. Conclusions

The present study afforded two novel triterpenoids from *C. pilosula.* Although they are not active towards SIRT1, they add new facets for the chemical profiling of *C. pilosula.*

## Figures and Tables

**Table 1:** ^1^H (600 MHz) and ^13^C-NMR (150 MHz) data of **1** and **2** in CDCl_3_ (*δ* in ppm, *J* in Hz).

No.	1		No.	2	
	*δ*_H_	*δ*_C_		*δ*_H_	*δ*_C_
1	5.63 (t-like, 7.7)	123.6	1	7.41 (d, 9.7)	141.0
2	Ha: 3.20 (dd, 16.6, 8.4)	34.0	2	6.24 (d, 9.7)	112.9
	Hb: 3.08 (dd, 16.6, 6.9)		3		172.8
3		173.0	4		73.2
4		73.2	5	2.89 (overlap)	51.6
5	2.58 (dd, 11.9, 5.8)	51.2	6	Ha: 2.02 (m)	27.6
6	Ha: 1.97 (m)	25.7 *^a^*		Hb: 1.43 (m)	
	Hb: 1.47 (m)		7	Ha: 1.56 (m)	25.0
7	Ha: 1.16 (overlap)	25.8 *^a^*		Hb: 1.32 (m)	
	Hb: 1.10 (overlap)		8	Ha: 1.29 (m)	34.6
8	1.91 (m)	48.2		Hb: 0.70 (m)	
9		140.8	9		201.2
10		141.7	10		120.5
11	5.19 (t-like, 5.3)	120.4	11	Ha: 3.05 (dd, 15.6, 11.9)	36.5
12	Ha: 2.02 (m)	35.5		Hb: 2.43 (dd, 15.6, 8.8)	
	Hb: 1.71 (m)		12	Ha: 2.35 (m)	28.1
13		43.0		Hb: 1.85 (m)	
14		46.5	13		44.2
15	Ha: 1.20 (overlap)	38.3	14		48.6
	Hb: 1.81 (dd, 14.1, 10.9)		15	Ha: 1.77 (dd, 13.8, 11.0)	42.4
16	4.06 (td-like, 10.7, 5.3)	77.1		Hb: 1.23 (overlap)	
17	1.44 (t-like, 10.7)	53.7	16	4.05 (td, 10.4, 7.1)	76.5
18	0.70 (s)	16.1	17	1.45 (overlap)	55.0
19	Ha: 2.90 (overlap)	42.9	18	0.78 (s)	15.6
	Hb: 2.70 (d, 13.4)		19		159,9
20	2.05 (m)	30.0	20	2.11, (m)	30.4
21	0.88 (d, 6.2)	19.1	21	1.01, (d, 6.5)	20.3
22	Ha: 1.86 (m)	44.0	22	Ha: 1.84 (m)	44.6
	Hb: 1.37 (dd, 14.0, 11.5)			Hb: 1.47 (overlap)	
23		107.3	23		106.7
24	Ha: 2.37 (dd, 12.9, 8.5)	42.6	24	Ha: 2.39 (dd, 12.9, 8.5)	42.4
	Hb: 1.68 (dd, 12.9, 11.5)			Hb: 1.71 (dd, 12.9, 11.5)	
25	2.89 (overlap)	34.1	25	2.91 (overlap)	34.1
26		179.7	26		179.6
27	1.22 (d, 7.3)	14.8	27	1.23 (d, 7.2)	15.0
28	1.12, (s)	26.4	28	1.05 (s)	25.5
29	1.18 (s)	28.9	29	1.29 (s)	29.2
30	0.83 (s)	21.4	30	1.31 (s)	26.9
OCH_3_	3.61 (s)	51.7			

*^a^* Signals might be interchangeable.

## Supplementary Materials

The following are available online: Figure S1. ^1^H-NMR spectrum of **1** in CDCl_3_, Figure S2. ^13^C-NMR and DEPT spectra of **1** in CDCl_3_, Figure S3. HSQC spectrum of **1** in CDCl_3_, Figure S4. HMBC spectrum of **1** in CDCl_3_, Figure S5. ^1^H-^1^H COSY spectrum of **1** in CDCl_3_, Figure S6. ROESY spectrum of **1** in CDCl_3_, Figure S7. HRESIMS of **1**, Figure S8. ^1^H-NMR spectrum of **2** in CDCl_3_, Figure S9. ^13^C-NMR and DEPT spectra of **2** in CDCl_3_, Figure S10. HSQC spectrum of **2** in CDCl_3_, Figure S11. HMBC spectrum of **2** in CDCl_3_, Figure S12. ^1^H-^1^H COSY spectrum of **2** in CDCl_3_, Figure S13. ROESY spectrum of **2** in CDCl_3_, Figure S14. HREIMS of **2**.
