# Supplementary material for: Two New Triterpenoids from the Roots of Codonopsis pilosula
Source: Molecules. 2018 Feb 11;23(2):0. doi: 10.3390/molecules23020383 (PMC6017360; doi:10.3390/molecules23020383)
Supplement: Supplementary file 1 [file molecules-23-00383-s001.pdf]

**Supplementary Materials**

# Two new triterpenoids from the roots of *Codonopsis pilosula*

Tao Zheng, Li-Zhi Cheng , Yong-Ming Yan, Fu-Ying Qin, Fu-Rong Xu and

Yong-Xian Cheng

Figure S1. $^{1}$H NMR spectrum of **1** in $CDCl_3$

Figure S2. $^{13}$C NMR and DEPT spectra of **1** in $CDCl_3$

Figure S3. HSQC spectrum of **1** in $CDCl_3$

Figure S4. HMBC spectrum of **1** in $CDCl_3$

Figure S5. $^{1}$H-$^{1}$H COSY spectrum of **1** in $CDCl_3$

Figure S6. ROESY spectrum of **1** in $CDCl_3$

Figure S7. HRESIMS of **1**

Figure S8. $^{1}$H NMR spectrum of **2** in $CDCl_3$

Figure S9. $^{13}$C NMR and DEPT spectra of **2** in $CDCl_3$

Figure S10. HSQC spectrum of **2** in $CDCl_3$

Figure S11. HMBC spectrum of **2** in $CDCl_3$

Figure S12. $^{1}$H-$^{1}$H COSY spectrum of **2** in $CDCl_3$

Figure S13. ROESY spectrum of **2** in $CDCl_3$

Figure S14. HREIMS of **2**

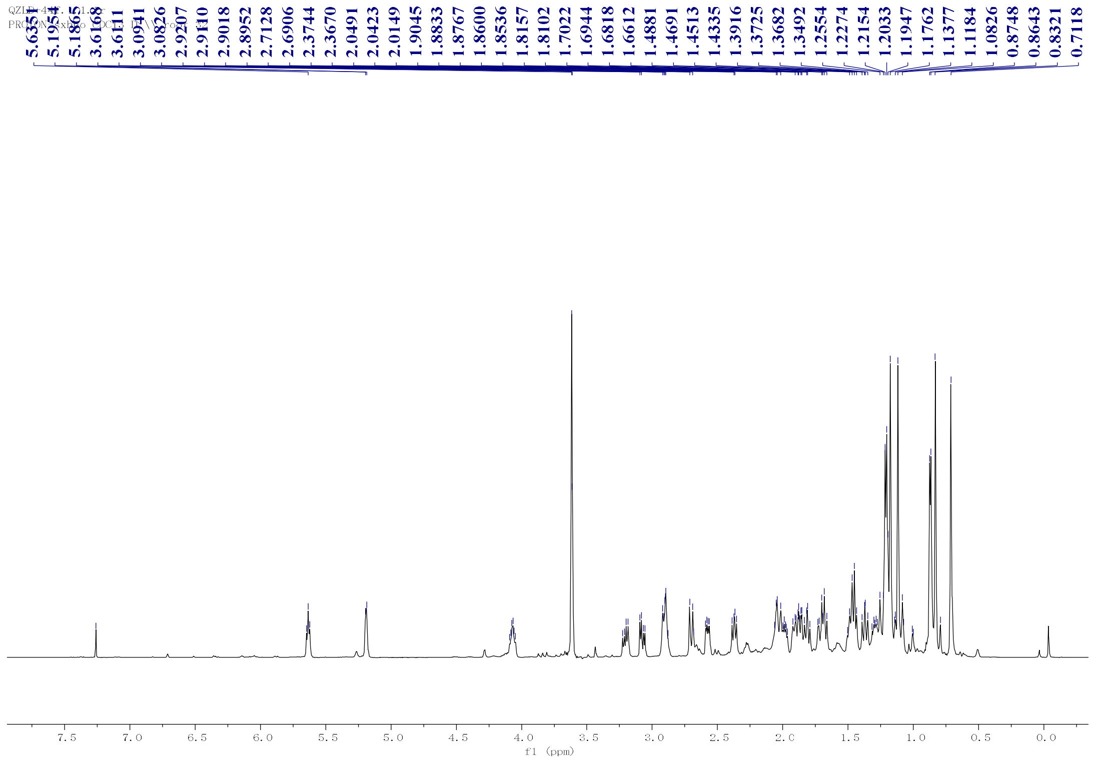

Figure S1. $^{1}H$ NMR spectrum of **1** in $CDCl_3$

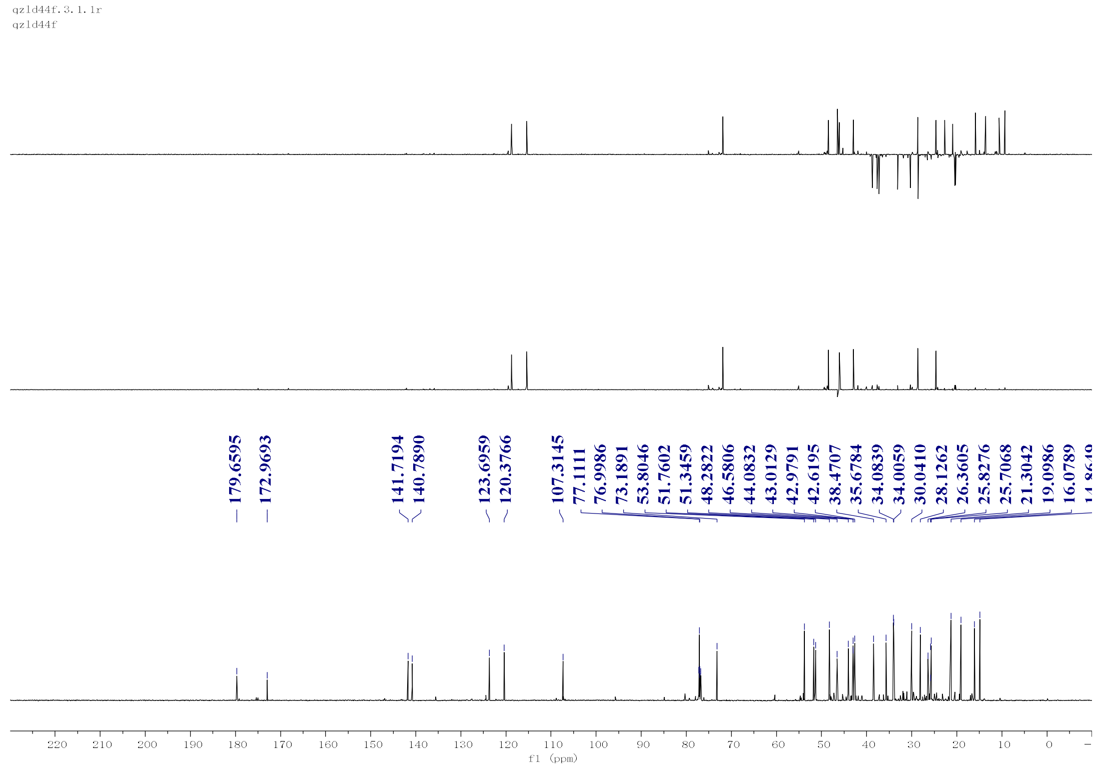

Figure S2. $^{13}C$ NMR and DEPT spectra of **1** in $CDCl_3$

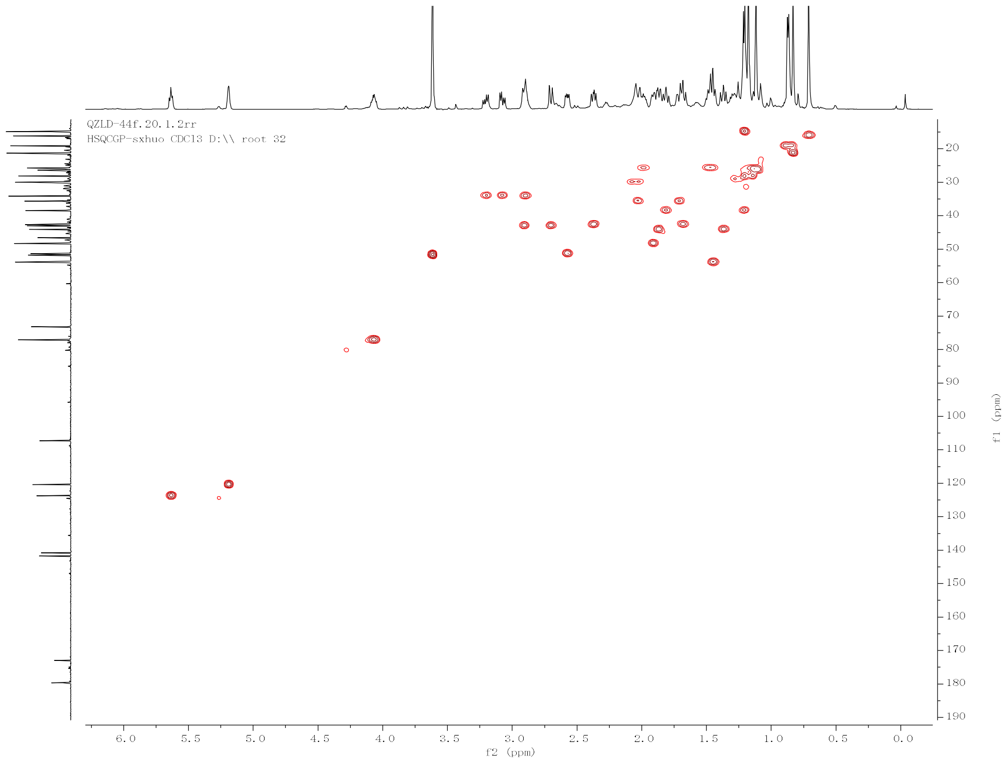


Figure S3. HSQC spectrum of **1** in $CDCl_3$

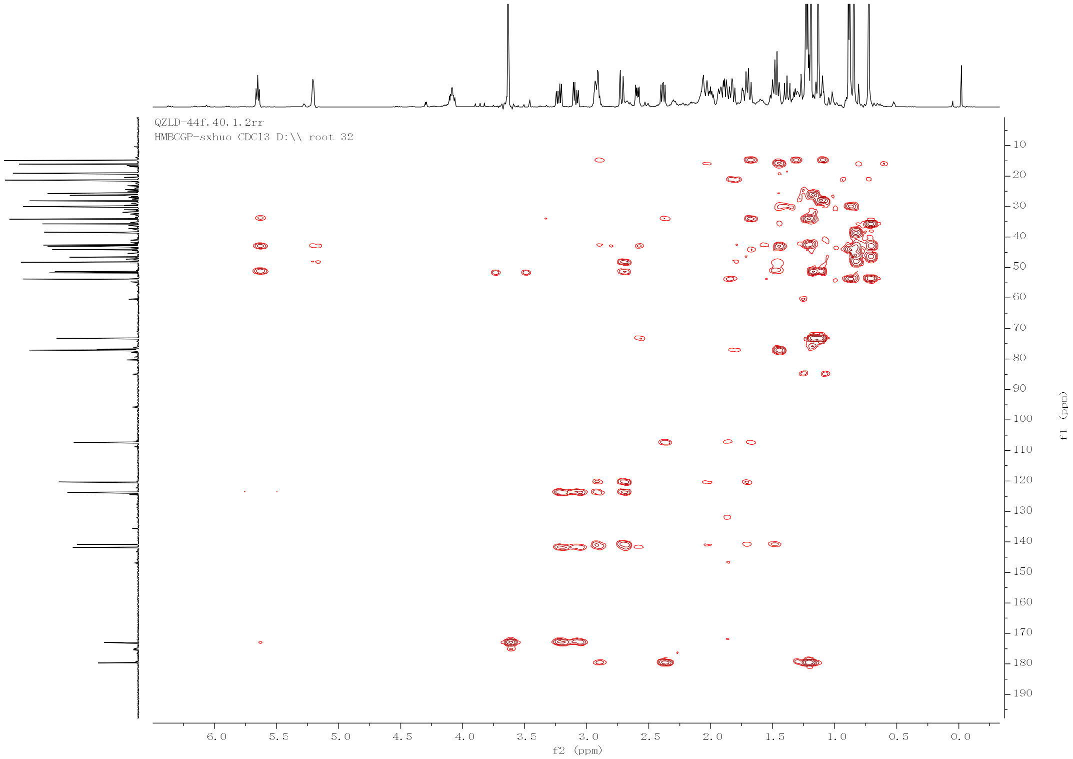


Figure S4. HMBC spectrum of **1** in $CDCl_3$

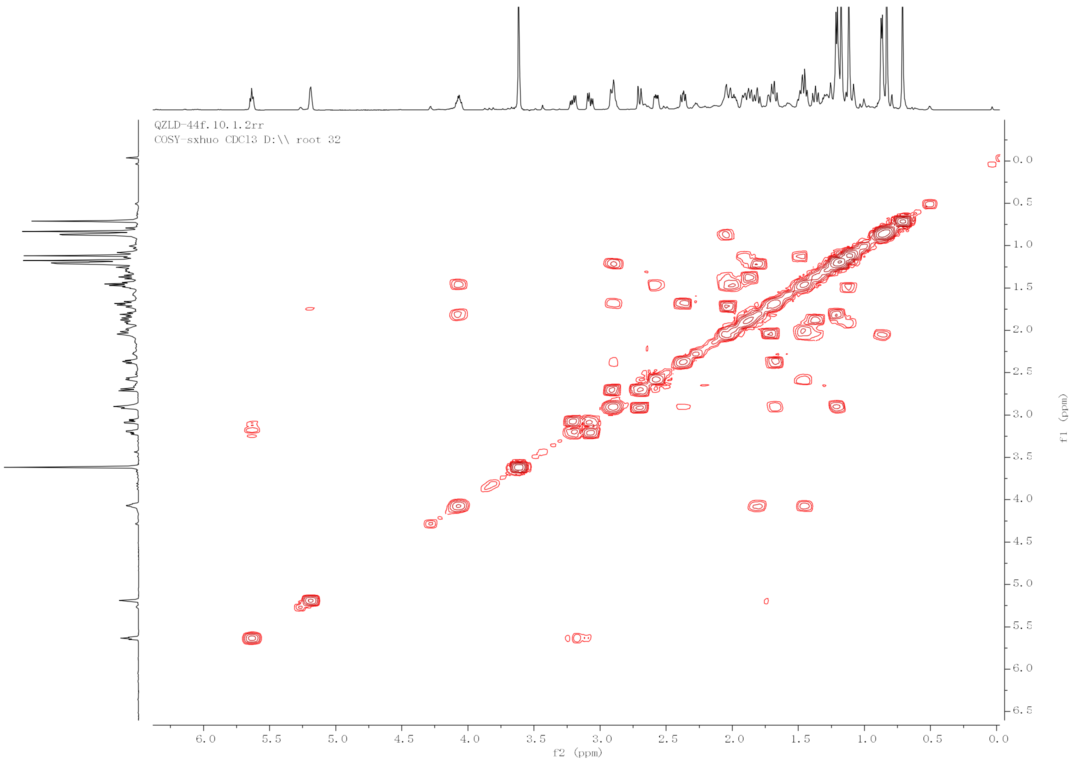


Figure S5. $^{1}$H-$^{1}$H COSY spectrum of **1** in $CDCl_3$

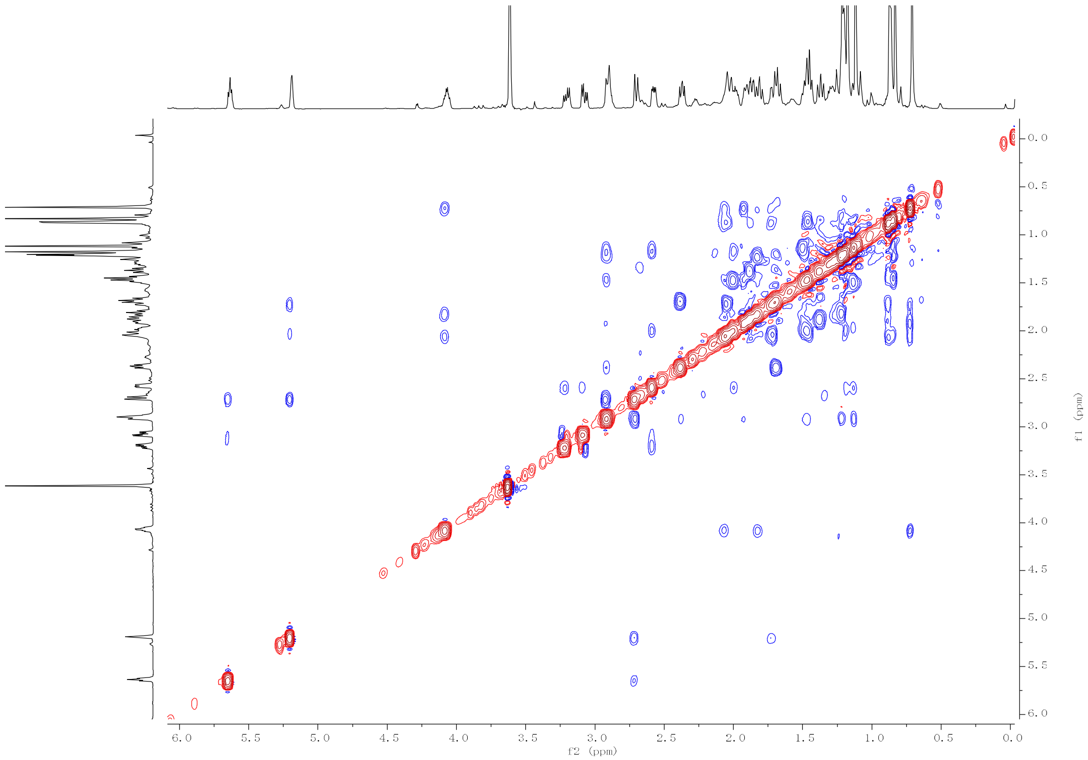


Figure S6. ROESY spectrum of **1** in $CDCl_3$

# Qualitative Analysis Report

| | | | |
|---|---|---|---|
| **Data Filename** | QZLD-44f.d | **Sample Name** | QZLD-44f |
| **Sample Type** | Sample | **Position** | P1-F8 |
| **Instrument Name** | Instrument 1 | **User Name** | |
| **Acq Method** | s.m | **Acquired Time** | 3/21/2017 4 |
| **IRM Calibration Status** | Success | **DA Method** | sibu.m |
| **Comment** | | | |

**Sample Group** **Info.**

**Acquisition SW** 6200 series TOF/6500 series
**Version** Q-TOF B.05.01 (B5125.2)

## User Spectra

| Fragmentor Voltage | Collision Energy | Ionization Mode |
|---|---|---|
| 135 | 0 | ESI |

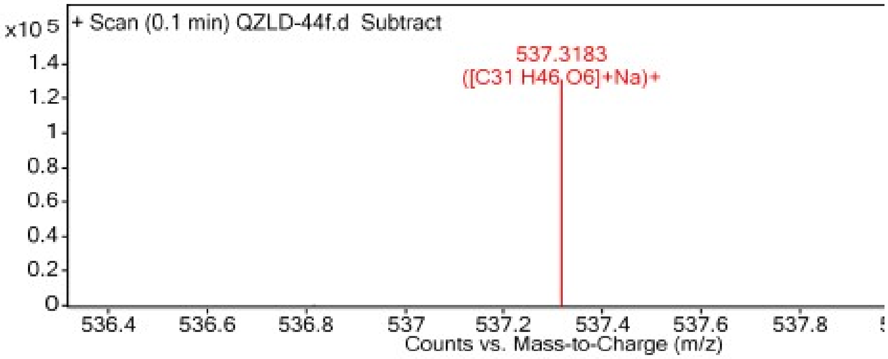


**Peak List**

| *m/z* | z | Abund | Formula | Ion |
|---|---|---|---|---|
| 537.3183 | 1 | 130682.29 | C31 H46 O6 | (M+Na)+ |
| 538.3211 | 1 | 42496.25 | C31 H46 O6 | (M+Na)+ |
| 553.2933 | 1 | 118959.99 | | |
| 554.2969 | 1 | 38942.48 | | |
| 555.3007 | 1 | 21362.3 | | |
| 641.3756 | 1 | 28081.68 | | |
| [illegible] | 1 | [illegible] | | |

Figure S7. HRESIMS of **1**

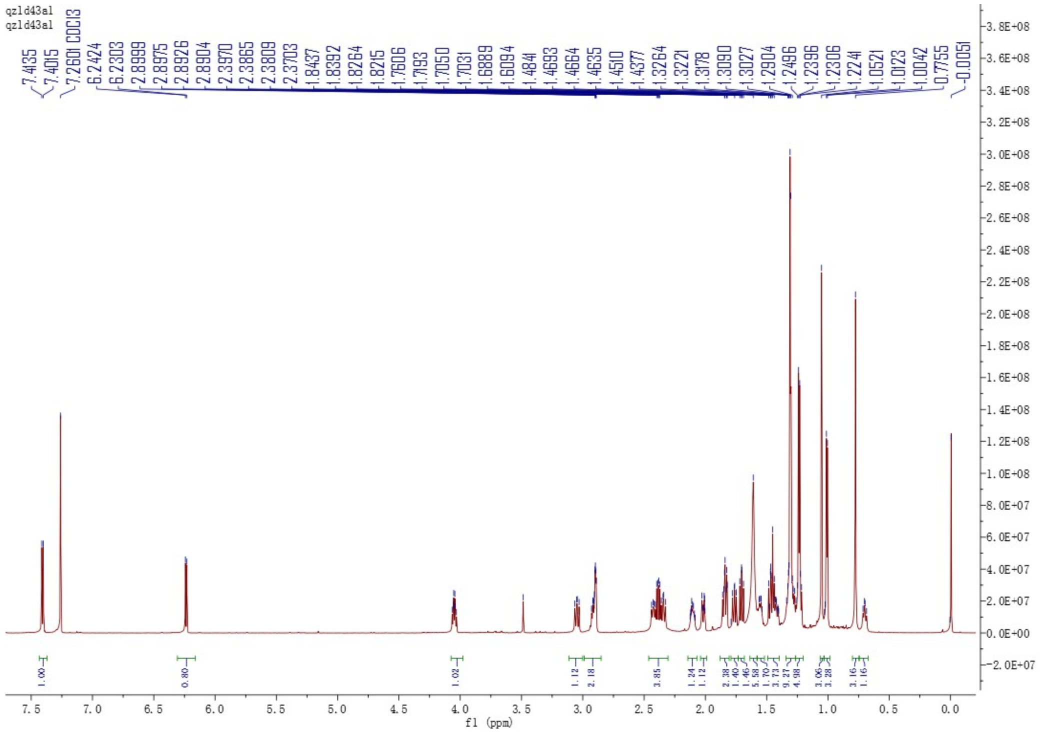


Figure S8. $^{1}$H NMR spectrum of **2** in $CDCl_3$

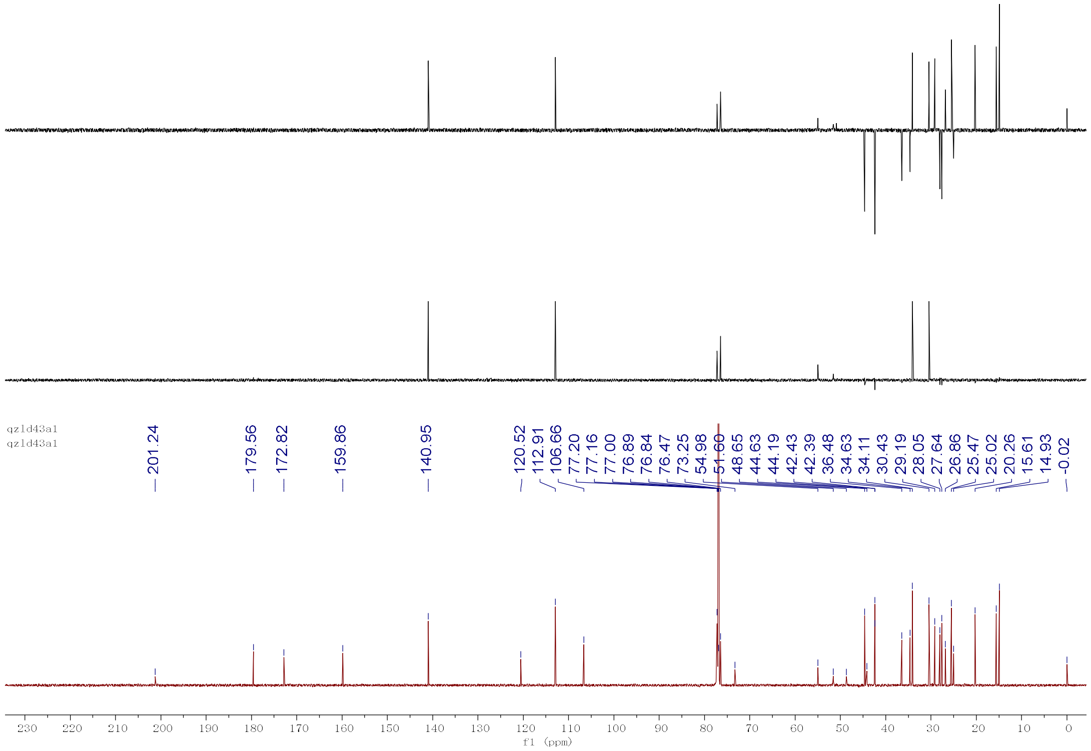


Figure S9. $^{13}$C NMR and DEPT spectra of **2** in $CDCl_3$

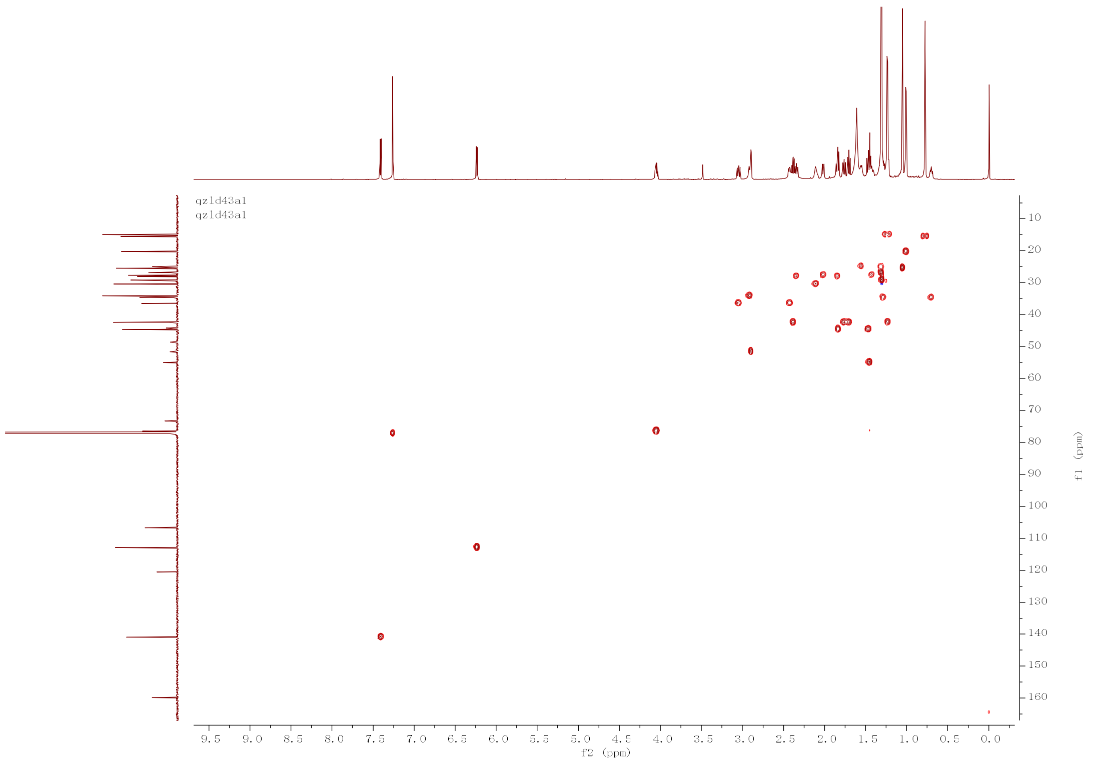

Figure S10. HSQC spectrum of **2** in $CDCl_3$

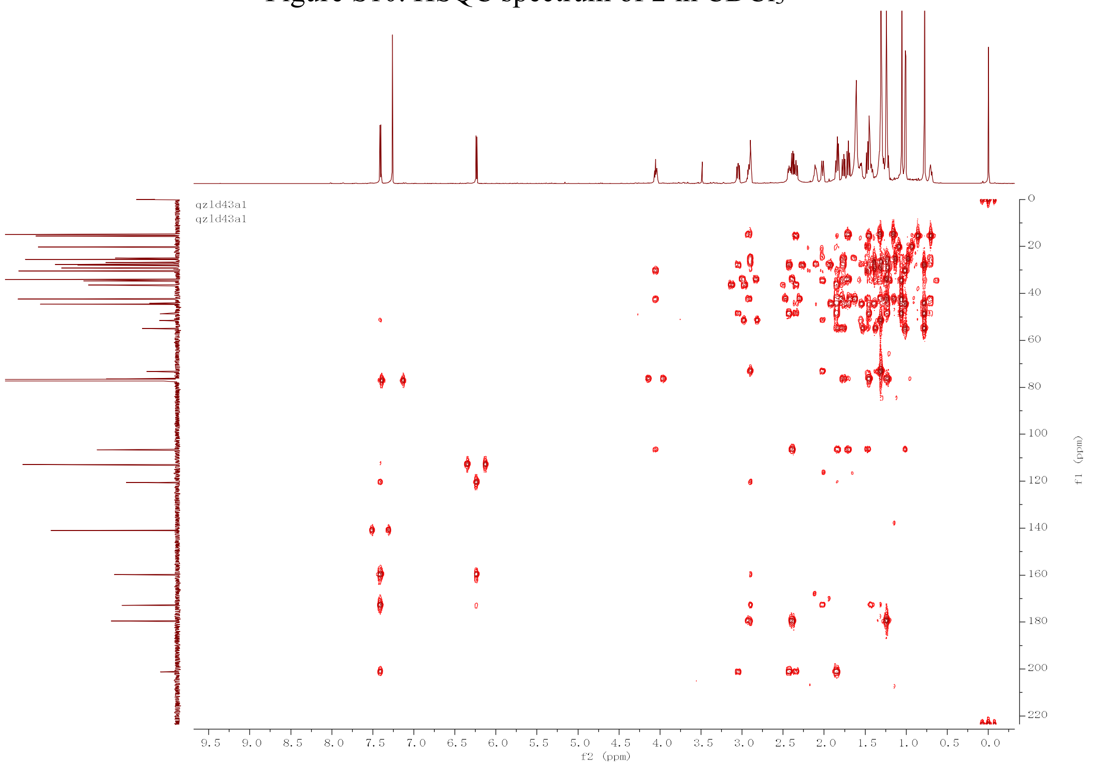

Figure S11. HMBC spectrum of **2** in $CDCl_3$

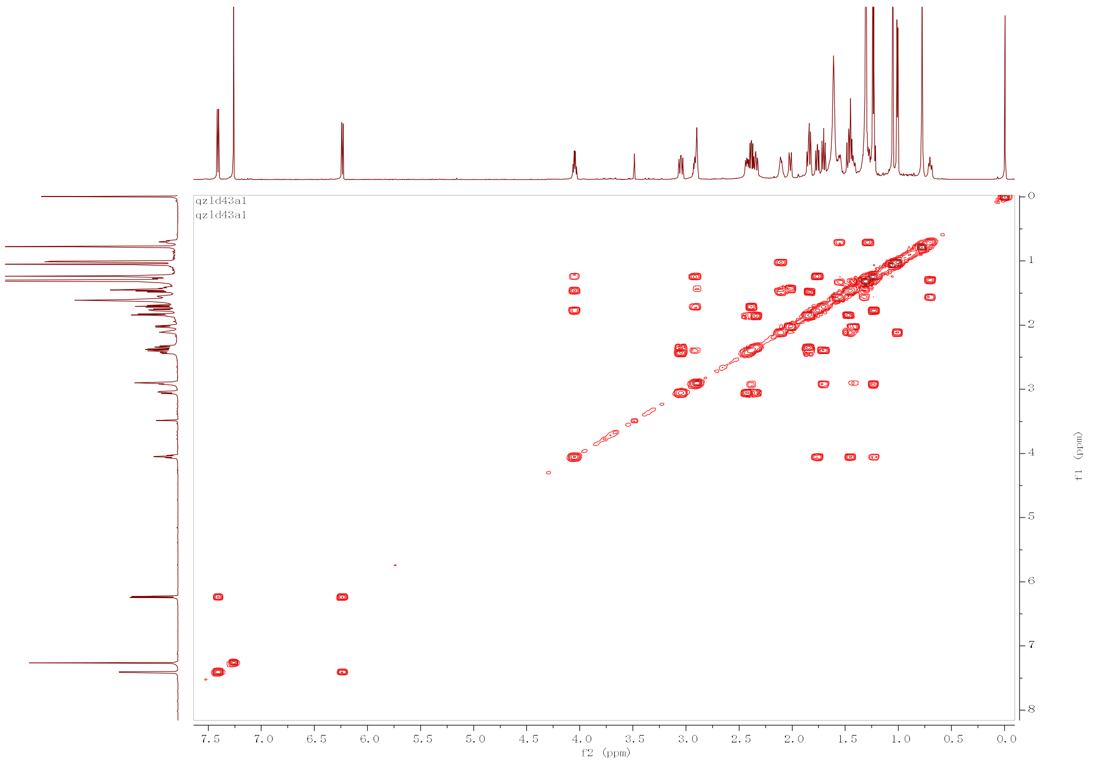


Figure S12. $^{1}H$-$^{1}H$ COSY spectrum of **2** in $CDCl_3$

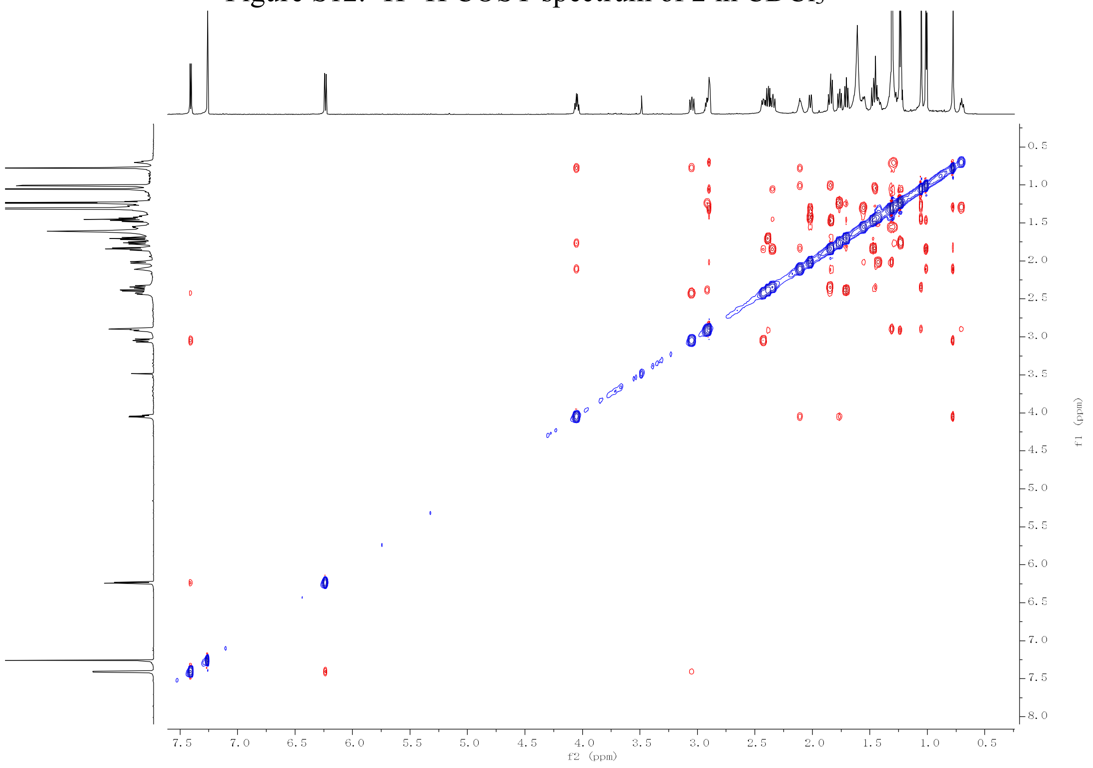


Figure S13. ROESY spectrum of **2** in $CDCl_3$

# Qualitative Analysis Report

| | | | |
|---|---|---|---|
| **Data Filename** | QZLD-43a1.d | **Sample Name** | QZLD-43a1 |
| **Sample Type** | Sample | **Position** | P1-F7 |
| **Instrument Name** | Instrument 1 | **User Name** | |
| **Acq Method** | s.m | **Acquired Time** | 3/21/2017 4:0[illegible] |
| **IRM Calibration Status** | Success | **DA Method** | sibu.m |
| **Comment** | | | |

**Sample Group** **Info.**

**Acquisition SW Version** 6200 series TOF/6500 series Q-TOF B.05.01 (B5125.2)

## User Spectra

| Fragmentor Voltage | Collision Energy | Ionization Mode |
|---|---|---|
| 135 | 0 | ESI |

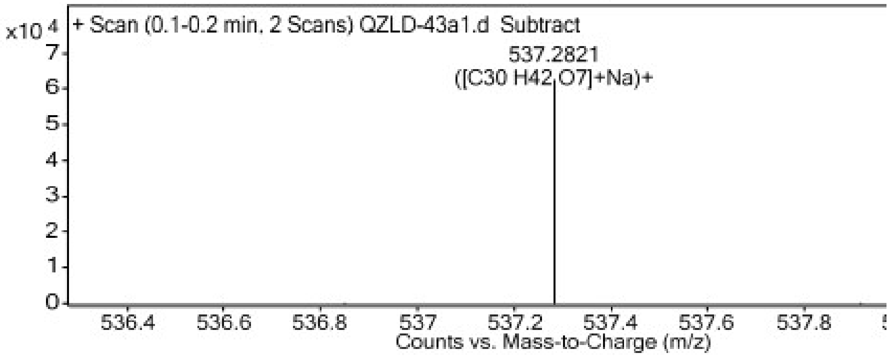


**Peak List**

| *m/z* | z | Abund | Formula | Ion |
|---|---|---|---|---|
| 515.2994 | 1 | 20157.2 | | |
| 537.2821 | 1 | 62447.47 | C30 H42 O7 | (M+Na)+ |
| 538.2851 | 1 | 19204.2 | C30 H42 O7 | (M+Na)+ |
| 553.2561 | 1 | 96512.36 | | |
| 554.259 | 1 | 30618.63 | | |
| 555.26 | 1 | 13423.11 | | |
| 641.3398 | 1 | 15743.22 | | |

Figure S14. HREIMS of **2**
